# Comparison of Dissolution and Surface Reactions Between Calcite and Aragonite in L-Glutamic and L-Aspartic Acid Solutions

**DOI:** 10.3390/molecules15010258

**Published:** 2010-01-11

**Authors:** Miyoung Ryu, Hwan Kim, Mihee Lim, Kwangsuk You, Jiwhan Ahn

**Affiliations:** 1 Korea Institute of Geoscience and Mineral Resources, 92 Gahwangno, Yuseung-gu, Daejeon, 305-350, Korea; E-Mails: miyung1@snu.ac.kr (M.R); limmh@paran.com (M.L); yks-30@hanmail.net (K.Y); ahnjw@kigam.re.kr (J.A); 2 School of Materials Science and Engineering, Seoul National University, Sillim-dong, Gwanak-gu, Seoul, 151-742, Korea; E-Mail: hwan94@snu.ac.kr (H.K)

**Keywords:** calcite, aragonite, dissolution, adsorption, amino acid solution

## Abstract

We have investigated dissolution and surface reaction of calcite and aragonite in amino acid solutions of L-glutamic (L-glu) and L-aspartic acid (L-asp) at weak acidity of above pH 3. The surface reactions of calcite and aragonite were related with the dissolution. Calcite was dissolved in both solutions but the dissolution was limited by an adsorption of Ca-carboxylate salt. Aragonite was neither dissolved nor reacted in amino acid solutions because the crystal surface consisted of a hard to dissolve structure.

## 1. Introduction

The dissolution of calcium carbonate has been researched extensively for several decades because calcium carbonate plays an important role in carbonate buffering in natural water. The dissolution kinetics of calcium carbonate is related with the neutralization of acidic lakes, weathering processes and elimination of scales in petroleum wells [1,2,3,4,5]. Calcite is the most stable phase among the various calcium carbonate polymorphs (calcite, aragonite and vaterite), so that it has received the most attention from many researchers [6,7,8] and therefore, the dissolution kinetics of calcite has been widely studied, but those of other phases have not been sufficiently studied yet. 

In research on the dissolution of calcite in weak and strong acid solutions, the initial pH of the solution was reported to be a critical factor in the determination of the dissolution rate [4,9]. In acetic acid solution, the dissolution of calcite occurred by the transport of reactant to the surface, surface reaction, and the transport of product from the surface. At pH values of below 2.9, the transport rates of reactant and product affected the dissolution rate, and at pH of above 3.7, the surface reaction rate also acted as a critical factor [9]. In nitric acid solutions, the initial pH was also reported as a determinant. The dissolution amount of calcite increased drastically at the pH of below 2, but the solution acidity did not affect the leaching of calcite above pH 3 [4].

Plummer *et al*. [10] have studied about the dissolution reaction of calcite and reported three mechanisms according to pH range. At the pH of less than 3.5, the dissolution rate was directly proportional to the hydrogen ion activity and was unrelated with the P_CO2_. In the pH region from 3.5 to 5.5, the dissolution rate depended on both pH and P_CO2_, so that the H_2_CO_3_ concentration was a determinant. Above pH 6.5, H_2_O was involved in the dissolution reaction. The three dissolution mechanisms are shown in the following reactions (1)–(3):

CaCO_3_ + H^+^ ↔ Ca^2+^ + HCO_3_^-^       -------- pH < 3.5   (1)

CaCO_3_ + H_2_CO_3_^0^ ↔ Ca^2+^ + 2HCO_3_^-^    -------- 3.5 < pH < 5.5 (2)

CaCO_3_ + H_2_O ↔ Ca^2+^ + HCO_3_^-^ + OH^- ^    -------- pH > 6.5   (3)

The dissolution kinetics of calcite have been studied with various additives in the solutions such as chelating agents, sodium dichromate, and polymaleic acid, phosphoric acid, or oxalic acid [9,11,12]. In the presence of calcium chelating agents, calcite was dissolved even at pH 12. The hydrogen ion attack of equation (1) did not occur, but the dissolution of calcite happened with the surface reaction between the chelating agent and calcite. The Ca chelating agent was adsorbed and a Ca-complex was formed on the surface of calcite. The Ca-complex resulted in weakening of the bonds between calcium and carbonate, and desorption of the Ca-complex occurred at pH 12. Therefore, calcite could be dissolved in a high pH region [9]. In the neutral pH region between 6 and 7, calcite was dissolved and the dissolution rate was affected by the presence of carbon dioxide, which retards its dissolution [11]. When the surface of calcite was pretreated by acidic solutions such as polymaleic, phosphoric and oxalic acids, the dissolution of calcite in acidic solutions was limited because the surface coating hindered the dissolution reaction on the surface [12].

Among calcium carbonate polymorphs aragonite represents a meta-stable phase and is found in certain living organisms such as oysters or abalones [13]. There are some results concerning the synthesis of aragonite using additives, but the dissolution of aragonite has been less investigated because of its instability. It has been reported that the dissolution of aragonite was controlled by the diffusion in the bulk of the solution and was determined by the surface area [14,5]. All of the previous research was focused on the dissolution of natural aragonite and bivalves in strong acid solutions, thus the dissolution behavior of synthetic aragonite also needs to be studied.

The present study aims to compare dissolution and surface reactions of synthesized calcite and aragonite in amino acids such as L-aspartic (L-asp) and L-glutamic (L-glu) acid. It is well known that the dissolution behavior of calcite depends on the surface reaction in solution and in general, the carbonate mineral is hardly treated its surface with organic chemicals [12]. However, Demjén *et al.* [15] have reported that the calcite could be treated by the silane coupling agent containing an amino group. Based on the fact that the amino group has reactivity for the calcium carbonate surface, amino acids were chosen as the solvent in this research. Calcite and aragonite were synthesized as rhombohedra and needle-like particles, respectively, in our laboratory. Dissolution is related to surface characters, thus we analyzed filtrates and the surface of solids after reactions. 

## 2. Results and Discussion

### 2.1. Dissolution equilibrium of calcite in 0.02 M acetic acid solution

Ca concentrations after dissolution for four hours are described in Table 1, showing both concentrations in mg/L and molarity (M). The dissolution of calcite resulted from the hydrogen attack as shown in reaction (1) and the Ca concentration from dissolved calcite would be equal to a half of the initial concentration of the acidic solution by charge balancing. Calcite was dissolved well in 0.02 M acetic acid solution and the Ca concentration from dissolved calcite was then 360 mg/L. It was almost same as the concentration in mg/L converted from 0.01M of Ca and was also equal to a theoretic value determined by the formula shown in the reaction (1). Therefore, the reaction for four hours in which the dissolution reached equilibrium was thus determined as an optimal condition for completely achieving calcite dissolution.

### 2.2. Dissolution behavior of calcite in amino acid solutions

Calcite was dissolved less in amino acid solutions than in acetic acid solution as described in Table 1. When the initial concentration was 0.02 M, the Ca concentrations in L-asp and L-glu solutions were 102 mg/L and 124 mg/L, respectively, which could be converted to 0.0025 M and 0.003 M Ca. Ca concentrations in 0.02 M amino acid solutions were much lower than that in acetic acid solution under the same conditions. That is, the amount of calcite dissolved in amino acid solution did not reach theoretical values. In addition, those in other solutions were lower than theoretical values as represented in Table 1. That is, the dissolution of calcite in amino acid solutions was extremely inhibited in spite of the acidic conditions present.

**Figure 1:**
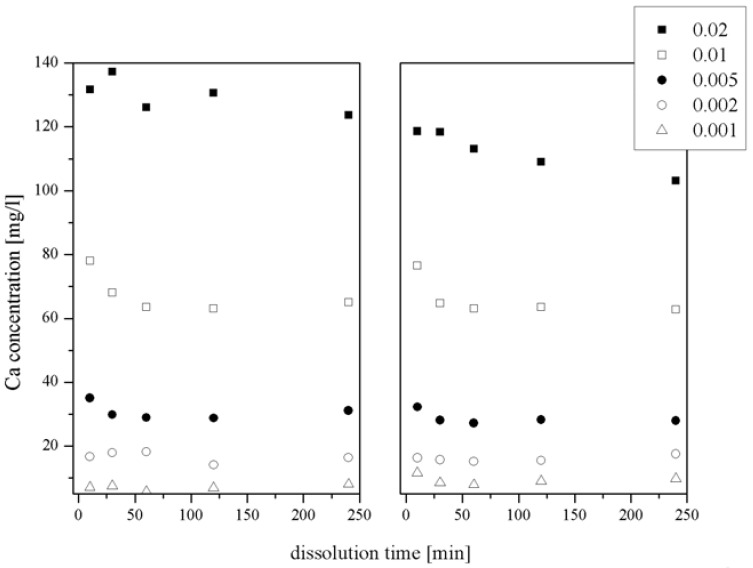
Ca concentrations in (a) L-glu and (b) L-asp solutions depending on the dissolution time. Overall Ca concentrations in (a) L-glu solutions were slightly higher overall compared with those in (b) L-asp solutions.

**Figure 2:**
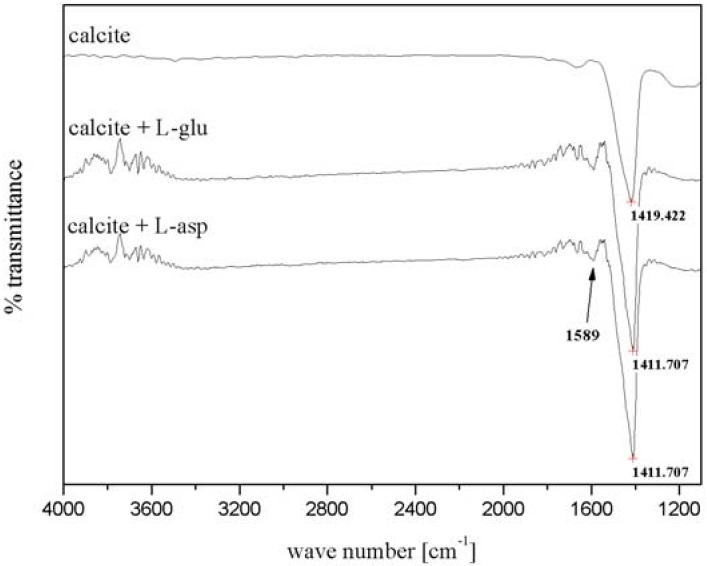
ATR-FTIR spectrums of calcite before and after of the dissolution. From the top, calcite, calcite dissolved in L-glu and calcite dissolved in L-asp.

The dissolution behavior of calcite according to the dissolution time was also investigated. Figure 2 shows Ca concentrations in L-asp and L-glu filtrates versus the dissolution time. Ca concentrations in L-glu solutions [Figure 1(a)] were slightly higher than those in L-asp solutions [Figure 1(b)], but those in both solutions were reduced with the dissolution time. After an hour, the dissolution reached an equilibrium state. From the reduction of Ca concentration in amino acid solution, we could consider that dissolved Ca ion adsorbed on calcite surface.

### 2.3. ATR-FTIR analysis of calcite surface

ATR-FTIR spectrums of calcite dissolved for four hours in L-glu and L-asp solutions are represented in Figure 2. After the dissolution, a carbonate adsorption band at 1,419 cm^-1^ shifted to 1,411 cm^-1^ and new absorption bands appeared near 3,600 cm^-1^ and 1,589 cm^-1^. The absorption bands appeared at about 3,500 cm^-1^ resulted from the existence of N-H bending vibrations [16]. Another band at 1,589 cm^-1^ and the shift of the peak from 1,419 cm^-1^ to 1,411 cm^-1^ could be explained by formation of a carboxylate salt. The characteristic pattern for carboxylate-metal ion complex (M-OOC-R) is the result of the appearance of two bands near 1,400 cm^-1^ and 1,600 cm^-1^ [16]. Accordingly, we could consider that a Ca-carboxylate salt existed on the surface of the calcite.

**Figure 3:**
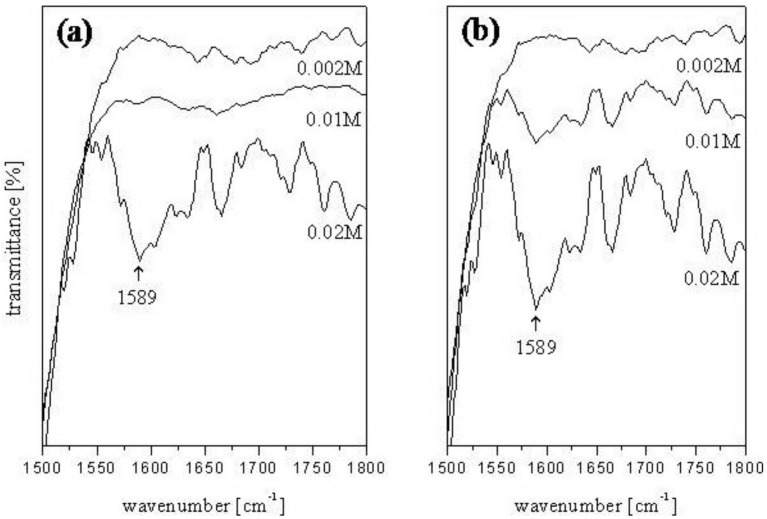
ATR-FTIR spectrums showing the carbonate stretching between 1,500 and 1,800 cm^-1^. From the top spectrum, the initial concentration of amino acid solution is 0.002, 0.01 and 0.02 M. (a) Calcite reacted with L-asp solutions and **(b)** reacted with L-glu solutions.

The carboxyl group reacts with the metal ions in solution and is adsorbed on the surface. The adsorption mode of carboxylate salt could be determined by the shape of IR adsorption band. If the carboxylate salt is adsorbed chemically on the surface, the adsorption band near 1,550 cm^-1^ appears as a single one; if it is adsorbed physically or is precipitated on the surface, the adsorption band between 1,500 and 1,600 cm^-1^ appears a doublet [17,18]. In the cases of calcite dissolved in 0.02 M amino acid solutions, a single adsorption band appeared at 1,589 cm^-1^, as shown in Figure 3. Therefore, we could consider that the Ca-carboxylate salt was chemically adsorbed on the surface. 

The Ca concentration dissolved from calcite reduced with decrease in the initial concentration. ATR-FTIR spectrums according to the initial concentration are shown in Figure 3. Absorption peaks at 1,589 cm^-1^ were intensified with an increase in the initial concentrations. The absorption peaks at 1,589 cm^-1^ were too weak to be detected on the surface of calcite reacted in 0.002 M of both amino acid solutions. When the calcite was dissolved in L-asp solution, the adsorption peak did not appear in 0.01 M solution but appeared in 0.02 M solution, as shown in Figure 3(a). In the case of calcite dissolved in L-glu solutions, however, the adsorption peak of Ca-carboxylate salt started appear from 0.01 M solution and became clearer with the increase to 0.02 M [see Figure 3(b)].

**Scheme 1:**
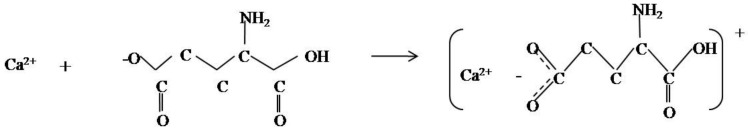
Chemical formula of the Ca-carboxylate salt formed by the reaction of Ca ion and L-glu ion.

From the Ca concentration and ATR-FTIR spectrum results, we could suppose that the adsorption of Ca-carboxylate salt on the surface was related with the dissolution of calcite. The amount of dissolved calcite increased with increasing initial concentration, and the adsorption peak of the Ca-carboxylate salt was also intensified. On the contrary, adsorption peaks related with Ca-carboxylate salt did not appear at low initial concentrations of both solutions. That is, Ca ion from calcite dissolution reacted with ionized amino acid and Ca carboxylate salt was formed as a final product, as shown in Scheme 1. And then, Ca-carboxylate salt was adsorbed on the surface of calcite. Consequently, the inhibition of calcite dissolution in amino acid solutions resulted from the adsorption of Ca-carboxylate salt.

### 2.4. Insoluble aragonite in amino acid solutions

Ca concentrations from aragonite dissolution in all acidic solutions involving 0.02 M acetic acid solution were below 10 mg/L, as represented in Table 1. That is, the needle-like aragonite was hardly dissolved in weak acidic solutions and reaction conditions such as pH, initial concentration and the reaction time did not affect its dissolution. Aragonite did not dissolve in amino acid solutions even after four days. 

**Figure 4:**
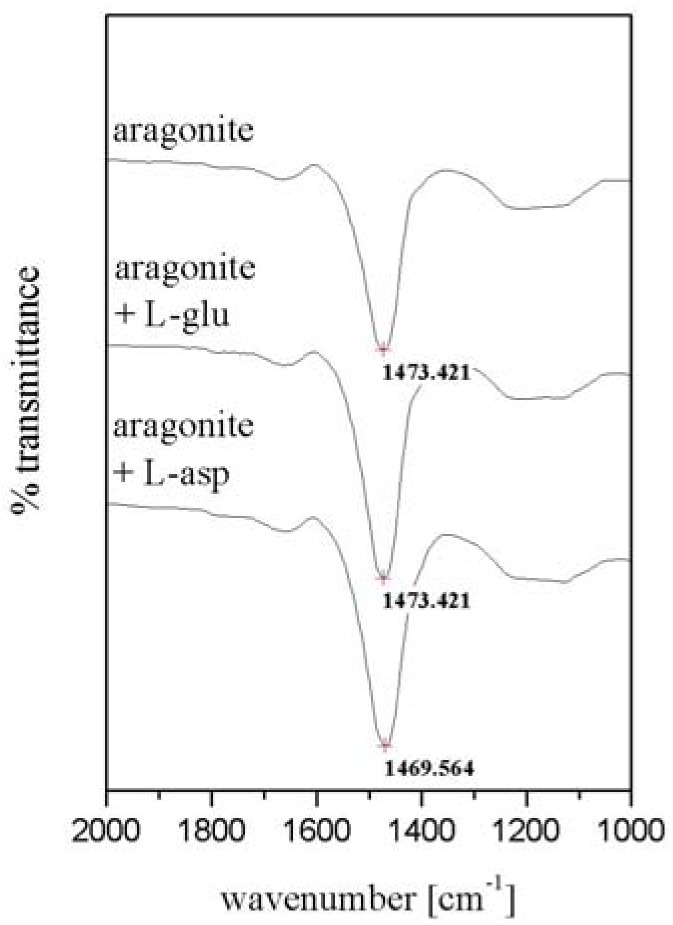
ATR-FTIR spectrums of aragonite. From the top, aragonite, aragonite dissolved in L-glu and aragonite dissolved in L-asp.

Because needle-like aragonite was not dissolved in acidic solutions, no Ca-carboxylate salt could be detected by ATR-FTIR, as shown in Figure 4. For the needle-like aragonite, a carbonate absorption peak appeared at 1,473 cm^-1^ but it does not shift, although the aragonite was reacted with 0.02 M amino acids for four hours. As aforementioned, the adsorption of Ca-carboxylate salt could occur after the Ca ion was dissolved from calcium carbonate. It is reasonable that the adsorption of Ca-carboxylate salt did not occur on the aragonite surface because aragonite was not dissolved at all.

**Figure 5:**
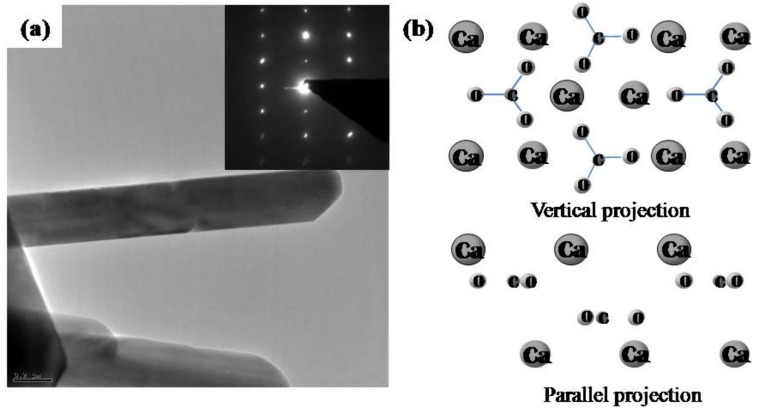
Transmission Electron Microscopy (TEM) image of needle-like aragonite and projection schemes of aragonite (010). (a) TEM image and Small Area Diffraction (SAD) pattern showing a single crystalline aragonite. (b) Vertical and parallel projection of aragonite (010).

The stability of the needle-like aragonite for the dissolution could be supposed to be the result of the crystal structure. Figure 5(a) shows TEM image and Small Area Diffraction pattern of the needle-like aragonite. One needle-like aragonite was composed of a single crystal and the lateral plane was calculated as (110) and (010) with SAD in Figure 5(a) [19]. Especially, aragonite (010) has been known as a cleavage plane. Vertical and parallel projections of aragonite (010) are shown in Figure 5(b). Ca atoms and CO_3_ groups are located at different layers along the [010] and the stacking sequence of layers is in the order of Ca-CO_3_-CO_3_-Ca-CO_3_ layers.

It was reported that the dissolution of calcium carbonate occurs with retention of charge balance [5]. Each layer of aragonite (010) is formed by a single charge such as Ca^2+^ or CO_3_^2-^, so that charge balance hardly occurs on the surface during the dissolution. That is, aragonite could not be continuously dissolved even though a little amount of aragonite was dissolved at the beginning of the reaction.

### 2.5. Surface morphology change due to the dissolution

**Figure 6:**
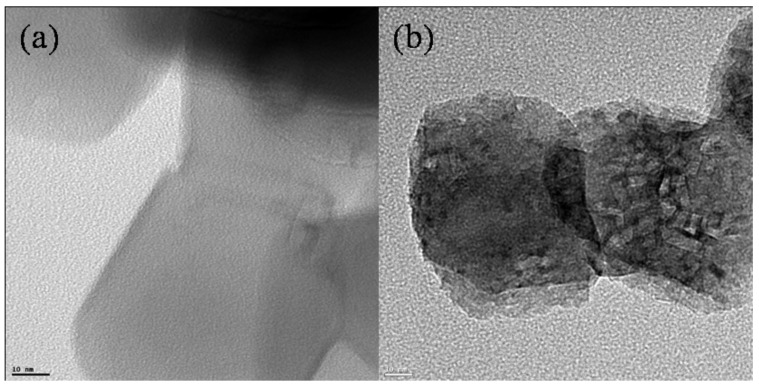
Surface morphology change of calcite.TEM images of (a) raw calcite surface and (b) dissolved calcite surface in 0.02 M L-glu solution for 4 hours.

**Figure 7:**
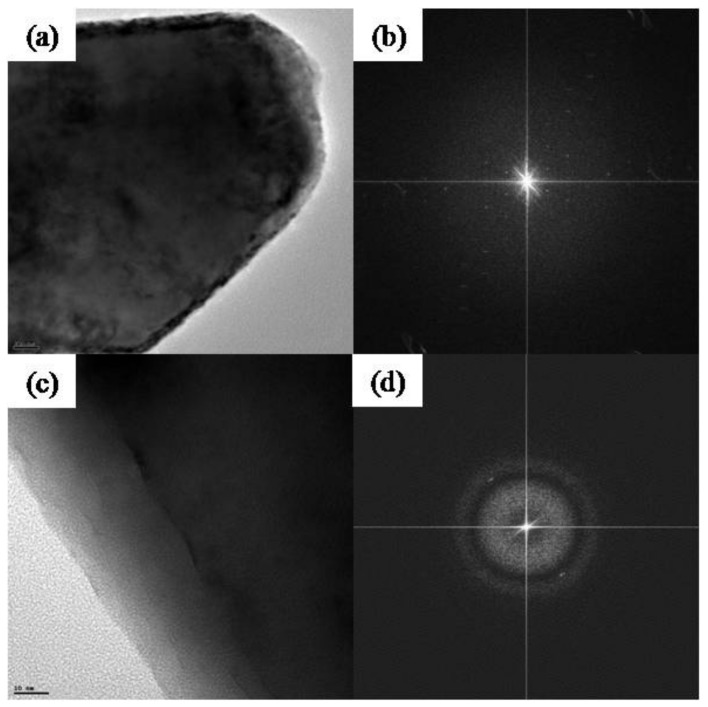
HRTEM and corresponding FFT of aragonite surface. (a) HRTEM image and (b) corresponding FFT before the dissolution of aragonite, and (c) HRTEM image and (d) corresponding FFT After the dissolution.

The surface morphology changes for the soluble calcite and the insoluble aragonite were different, as shown Figure 6 and Figure 7. Before the dissolution, the surfaces of calcite and aragonite were very clear and there were no defects or roughness on surfaces [see Figure 6(a) and Figure 7(a)]. After the dissolution in 0.02 M L-glu solution, dissolution pits were formed on calcite surface however they did not appear on the aragonite surface [see Figure 6(b) and Figure 7(b)]. During the dissolution of calcite, the shape of rhombic pit could appear on the surface due to its crystal symmetry [2]. In Figure 6(b), we could see the rhombic pits which could be regarded as surface defects formed by the dissolution. Though no surface defects were observed on the surface of aragonite, as shown in Figure 7(c), we could note a surface crystallinity change. Crystallinity of the particle can be confirmed with Fast Fourier Transformation (FFT) calculation of HRTEM image as shown in Figures 7(b) and (d). When the HRTEM image of crystalline particle was transformed to FFT mode, we could observe spots in FFT of Figure 7(b). After the dissolution, a bright circle appeared in FFT mode which indicated non-crystalline phase was formed on the surface as in Figure 7(d). The surface of aragonite could be stable on the dissolution in acid solution however the surface crystallinity could not be kept during the dissolution. With this research, we have not defined the reason for the surface change and this needs to be studied more in the future work.

## 3. Experimental

### 3.1. Raw materials

Calcium carbonates were synthesized in our laboratory. Calcite was prepared by a carbonation method which is synthetic process that involves blowing CO_2_ gas into a Ca(OH)_2_ slurry at room temperature. The initial concentration of Ca(OH)_2_ slurry was 0.2 M and the injection rate of CO_2_ gas was 50 mL/min. The carbonation process was determined by a pH change from pH 12.4 to 6.8. The reaction was finished with the pH of the solution was below 6.8 [20]. Synthesized calcite was a single phase and its specific surface area was 12 m^2^/g. Aragonite was synthesized with Na_2_CO_3_ and Ca(OH)_2_ solution at 80 °C and NaOH solution was used to control a dissolution rate of Ca(OH)_2_. Ca(OH)_2_ (0.5 mole) was mixed in 2.5 M NaOH solution (400 mL) and 0.5 M Na_2_CO_3_ solution (600 mL) was injected into the Ca(OH)_2_-NaOH solution at 30 mL/min. Synthesized aragonite was also a single phase and its specific surface area was 2 m^2^/g. 

**Figure 8:**
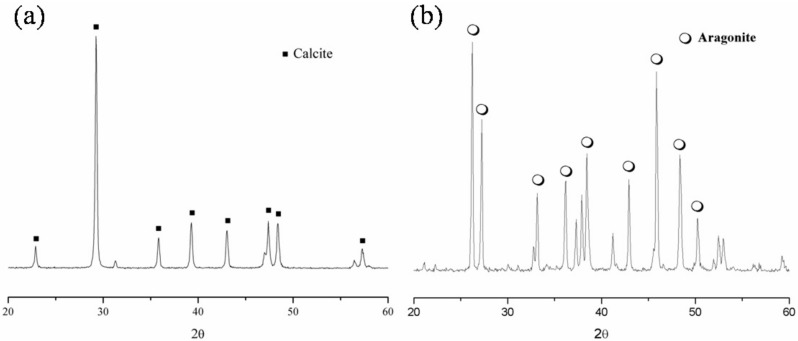
XRD patterns of synthesized calcite and aragonite. (a) Rhombohedral calcite and (b) needle-like aragonite.

**Figure 9:**
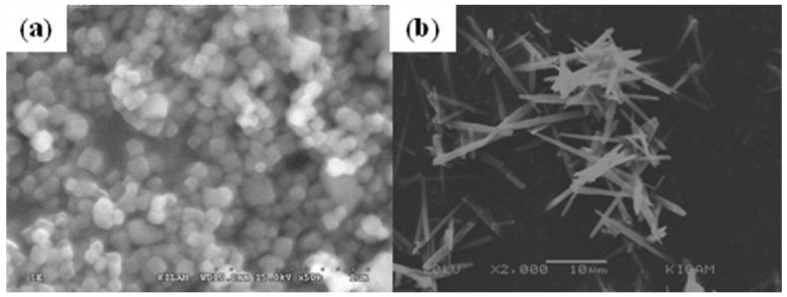
SEM images of synthesized calcite and aragonite. (a) Rhombohedral calcite of less than 100 nm and (b) needle-like aragonite.

Synthesized calcite and aragonite were composed of a single phase and unreacted materials or second phase was not detected in XRD analysis as shown in Figures 8 (a) and (b). Synthesized calcite and aragonite were observed by the analysis of Scanning Electron Microscopy (SEM, JSM 6300, JEOL; FESEM, S-4800, HITACHI) as represented in Figure 9. The particle size of calcite was just below 1 μm and the shape appreared as rounded rhombohedra as in Figure 1(a), whereas that of aragonite was very large and the length of the major axis was even above 10 μm and that of the minor axis was almost 1 μm [Figure 1(b)]. In order to analyze the surface morphology, Transmission Electron Microscopy (TEM, F20, Tecnai) image was taken, and Small Area Diffraction (SAD) was analyzed.

Acidic solutions were prepared using amino acids containing acidic side chains. The chosen amino acids were L-aspartic (L-asp; HO_2_CCH(NH_2_)CH_2_CO_2_H) and L-glutamic (L-glu; HO_2_C(CH_2_)_2_(NH_2_)CH_2_CO_2_H) acids which were ionized in aqueous solution, and solutions of pH 3.1 and pH 3.4 were formed. Acid solutions of amino acids were made to be 0.002, 0.005, 0.01 and 0.02 M, respectively. In order to compare the dissolution behavior of calcium carbonate in amino acid solutions and general weak acid solution, 0.02 M of acetic acid solution was used.

### 3.2. Experimental set-up 

The dissolution reaction was performed using a one liter glass vessel and a mechanical stirrer (RW 20 DZM, IKA) equipped with a two-bladed Teflon propeller type impeller. Either calcite or aragonite (15 g) was added into each acid solution (250 mL) as aforementioned. The agitation rate was fixed at 250 rpm and the dissolution was carried out at room temperature. Aliquots (40 mL each) were taken out from the suspensions at 10 min, 30 min, 1 hour, 2 hour and 4 hour during the dissolution and filtered using a membrane filter of 0.45 μm pore size. After the filtration, each solid residue on the filter paper was dried at 60 °C for a day to be used for its surface analysis and each filtrate was separately collected to be used for the analysis of its Ca concentration. Filtered solids were analyzed with an ATR-FTIR (Nicolet 380, Thermo Instrument) which is a method to detect the IR spectrum reflected from target materials. Ca concentration in the filtrate was measured using AAS (AA-6800, Shimadzu). A Ca calibration curve was then made by three standard solutions of 10, 20 and 40 mg/L.

## 4. Conclusions

Surface reactions of calcite and aragonite were quite different in amino acid solutions. Calcite was dissolved in amino acid solutions, whereas the adsorption of Ca-carboxylate salt limited the dissolution behavior. Aragonite did not dissolve in amino acid solutions and no adsorption of Ca-carboxylate salt appeared on its surface either. The lateral surface of needle-like aragonite consisted of (010) on which its charge balance was hardly kept during the dissolution. In the future study, it is necessary to investigate the surface morphology changes of calcite and aragonite caused by the dissolution. 

## Figures and Tables

**Table 1:** Ca concentration at the dissolution for four hours. Ca concentrations by the calcite dissolution depended on the initial concentration and acidic solutions. Ca molarity (M) of in each filtrate was described in parenthesis. Ca concentrations in dissolution filtrates of aragonite could be detected by AAS analysis.

	0.002 M	0.005 M	0.01 M	0.02 M
**Calcite + acetic acid**	-	-	-	360 mg/L (0.01 M)
**Calcite + L-asp**	13 mg/L (0.0003 M)	24 mg/L (0.0006 M)	60 mg/L (0.0015 M)	102 mg/L (0.0025 M)
**Calcite + L-glu**	16 mg/L (0.0004 M)	31 mg/L (0.0008 M)	65 mg/L (0.0016 M)	124 mg/L (0.0031 M)
**Aragonite + each of the solutions**	Under 10 mg/L			
